# Human-Centered Design and Benefit Realization Management in Digital Health Care Solution Development: Protocol for a Systematic Review

**DOI:** 10.2196/56125

**Published:** 2024-05-21

**Authors:** Kaisa Kauppinen, Pantea Keikhosrokiani, Sehrish Khan

**Affiliations:** 1 Faculty of Information Technology and Electrical Engineering University of Oulu Oulu Finland

**Keywords:** human-centered design, digital health care solution, electronic health record, benefit realization management, digital health care, health care software, digital health, information technology, IT, usability

## Abstract

**Background:**

Earlier research shows that a significant number of resources are wasted on software projects delivering less than the planned benefits. It has, however, been evidenced that adopting a human-centered design approach when designing health devices can be beneficial. This understanding from earlier research has raised our interest in investigating how human-centered design might contribute to realizing the potential benefits of health care software projects. To our current knowledge, this intersection of human-centered design and benefit realization management has not yet comprehensively and consistently been researched within the context of digital health care solutions. Therefore, there is a need for evidence synthesis using systematic reviews to address this potential research gap.

**Objective:**

The objective of this study is to examine if human-centered design helps benefit realization management processes in the development of digital health care solutions and thereby enables better benefit realization. We explore the evidence of assumed or confirmed benefits of using human-centered design in the health care domain and whether better results have been reported when the benefit realization management process is followed.

**Methods:**

This protocol was developed following the PRISMA-P (Preferred Reporting Items for Systematic Review and Meta-Analysis Protocols) guidelines. The proposed review would use a planned and systematic approach to identify, evaluate, and synthesize relevant and recent studies (reported in English) to see if there is evidence that using human-centered design and benefit realization management has a positive effect on realizing set benefits in those projects. We will commence a systematic literature search using human-centered design, benefit realization management, and health care–related search terms within 5 repositories (ACM Digital Library, PubMed Central, Scopus, PubMed, and Web of Science). After removing duplicate results, a preliminary scan for titles and abstracts will be done by at least 2 reviewers. Any incongruities regarding whether to include articles for full-text review will be resolved by a third reviewer based on the predefined criteria.

**Results:**

Initial queries of 2086 records have been executed and papers are being prescreened for inclusion. The search was initiated in December 2023 and the results are expected in 2024. We anticipate finding evidence of the use of human-centered design in the development of digital health care solutions. However, we expect evidence of benefitting from both human-centered design and benefit realization management in this context to be scarce.

**Conclusions:**

This protocol will guide the review of existing literature on the use of human-centered design and benefit realization management when developing digital health care solutions. The review will specifically focus on finding evidence of confirmed benefits derived from the use of human-centered design and benefit realization management. There may be an opportunity to gain a broader understanding of the tools or approaches that provide evidence of increased benefit realization within the health care domain.

**International Registered Report Identifier (IRRID):**

DERR1-10.2196/56125

## Introduction

### Background

Throughout the last decades, IT investments have grown significantly in all industries. Smaller portions of IT service providers´ budget**s** are often allocated to seeking improvements in their product or service development practices. Human-centered design (HCD) or user-centered design (UCD) belongs to this portion.

### Discussion on Human-Centered Design and User-Centered Design

British Standards Institution defines HCD as “an approach to interactive systems development that aims to make systems usable and useful by focusing on the users, their needs, and requirements, and by applying human factors/ergonomics, and usability knowledge and techniques” [1]. On the other hand, R Kling [2] states UCD is an approach that bases the design, testing, and development of a product or a service on the needs of the users affected by it. UCD can thereby be described as offering a set of principles and strategies to guide the design from the perspectives of (and with input from) those humans who eventually use that product or service. When discussing UCD in the context of digital health care solutions, “user” can refer to many different groups. A user in that context can mean a clinician (eg, a doctor or a nurse), other health care professionals, care givers, or a patient. Given that, it may be more appropriate to talk about HCD, rather than UCD in the health care context, since multiple user groups are of interest here, rather than just one. In addition, Walters [3] and Steen et al [4] differentiate UCD and HCD. They argue that HCD places more emphasis on different stakeholders’ varying needs and broader contexts. This definition aligns well with the different user groups we are interested in within the health care context, particularly those who use digital health care solutions.

### Digital Health Care Solution

By digital health care solutions, we mean a wide range of technologies used to improve the delivery, efficiency, and accessibility of health care services using digital tools and IT. Some examples of digital health care solutions include telehealth solutions (eg, video conferencing platforms for doctor-patient consultations), electronic health records (ie, electronic systems that store and manage patient health information), and mobile health apps (eg, medication reminder apps and mental health apps).

### On Benefits and Related Disciplines

As noted by earlier research [5-7], a significant number of resources are wasted on software projects delivering less than the planned benefits. Furthermore, Tursunvayeva et al [8] discuss how a range of sociotechnical challenges often hamper the benefit realization processes, with many expected improvements (or benefits) either not being realized or only partially realized. These studies also discuss benefits management (BM) and explain that BM is a topic of discussion due to overlapping disciplines within BM, such as benefit realization, benefit realization management (BRM), and value management. Looking back to earlier publications on BM, BM is described as the overall process of evaluating and realizing IT benefits. Ward et al [9,10], on the other hand, define BRM as “the process of organizing and managing such that potential benefits arising from the use of IT are actually realized.” In addition, BRM can be described as a comprehensive management idea. The definition of BRM usually focuses on either the benefits lifecycle or the realization of potential investment benefits in change. One often used definition of BRM is “the process of organizing and managing, so that potential benefits, arising from investment in change, are actually achieved” [11]. Tursunbayeva et al [8] also discuss how benefits can be divided into 2 groups when associated with IT projects—expected benefits and realized benefits. The former can have an important role in shaping the enablement of the latter.

Health care professionals have been studied regarding their adaptation to health care technology, and the study showed that technological anxiety had a negative influence on behavioral intention [12]. Furthermore, as noted by Persson [13] in her study on technology-centered versus human-centered perspective in the design and development process, reducing the focus on technical advancements in favor of the needs of the health care user community was found to be favorable. It has also been evidenced that taking an HCD approach when designing health devices, particularly for older adults, can be beneficial and can increase the likelihood of technology acceptance [14]. Consequently, earlier research has shown that planned benefits can be misleading in digital transformation projects unless they are consistently followed up regularly throughout the process.

### Establishment of Interest and Consideration

This understanding from earlier research has raised our interest in investigating how HCD might contribute to organizing, managing, and realizing the planned benefits within health care software projects. To our current knowledge, this intersection of HCD and BRM has not yet been comprehensively and consistently researched within the context of health care IT. It is, however, recognized that benefits may not appear as a direct consequence of the implementation of a digital health care solution and those benefits are necessarily not financially measurable (eg, patient or clinician experience) [15]. It is worth noting that there may be multiple reasons for the translational gap in digital health care solution development. We, therefore, value understanding and providing evidence of whether there is already an established correlation and dialogue between design techniques and software development in realizing the targeted benefits (both quantitative and qualitative) in the health care context.

Toward the goal of successfully identifying, analyzing, and summarizing these existing studies, we aim to systematically review design and development approaches in the health care domain. This includes examining their design techniques, exploration of BRM, and capabilities to realize the planned benefits. The theoretical assumption of this study is that when HCD is used in digital health care solutions’ development projects, the set and planned benefits are better realized. Additionally, it will be controversially considered whether we can assume that a technology-centered approach in the health care sector has contributed to the realization of disbenefits.

### Research Questions

In this paper, we examine if HCD helps the BRM process in the development of digital health care solutions**,** thereby enabling better benefit realization.

To address our aims, we have set the following research questions:

- Is HCD used in the design and development of software solutions for health care domain? What are the assumed or confirmed benefits of HCD?
- Is BRM used in the process of developing health care software solutions? Have better results been reported when BRM is included in the process?
- Have there been studies on whether HCD helps the BRM process in the development of digital health care solutions? What is the evidence that planned benefits have been better realized with the help of HCD?

### Objectives

To answer our research questions, we have identified the following objectives for the study:

1. to explore the evidence of assumed or confirmed benefits of using HCD in the development of digital health care solutions.
2. to investigate whether there is evidence that better results have been reported when BRM is included in the development process of digital health care solutions.
3. to identify whether there is evidence that planned benefits have been better realized with the help of HCD and BRM in health care context.

## Methods

### Design of the Study

**Figure 1:**
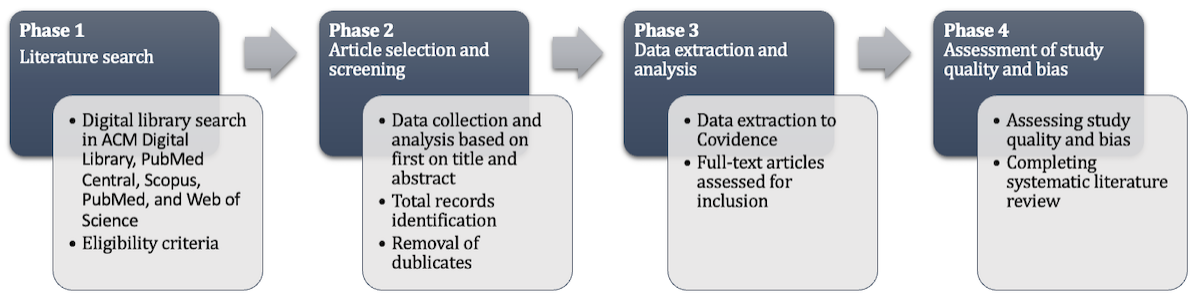
Illustration of the study structure.

For the design of the systematic review, we will use the PRISMA-P (Preferred Reporting Items for Systematic Review and Meta-Analysis Protocols) guidelines [16]. PRISMA-P consists of a literature search, article selection and screening, data extraction and analysis, as well as an assessment of study quality and bias. Figure 1 depicts the study structure, which is explained in more detail in the next sections.

### Literature Search

We will survey 5 large databases of digital literature—ACM Digital Library, PubMed Central, Scopus, PubMed, and Web of Science—using keywords and terms presented in Table 1.

Search strings need to be adapted to suit the specific requirements of the different databases and electronic libraries.

Search terms focus on 3 key areas: HCD terms (eg, human-centered and user-centered), BRM terms (eg, benefit management, benefit realization management, and benefit realization), and health care–related terms (eg, electronic health record and health care IT). An initial digital library search has provided 2086 papers.

**Table 1:** Used search terms for data collection.

Field of interest	Search string
Human-centered design and related terms (in first round, titles and abstracts are in focus)	“human-centered design” OR “human centered design” OR “human-centred design” OR “human centred design” OR “user-centered design” OR “user centered design” OR “user-centred design” OR “user centred design”
Benefit realization management and related terms (in first round, titles and abstracts are in focus)	“assumed benefits” OR “confirmed benefits” OR “benefit realisation management” OR “benefit realization management” OR “benefit realisation” OR “benefit realization” OR “benefit management”
Health care–related terms (in first round, titles and abstracts are in focus)	e-health OR ehealth OR “health information system” OR “healthcare information system” OR “hospital information system” OR “clinical information system*” OR “electronic health record” OR “digital health” OR “mobile health application” OR mhealth
Combined search strings	(“human-centered design” OR “human centered design” OR “human-centred design” OR “human centred design” OR “user-centered design” OR “user centered design” OR “user-centred design” OR “user centred design”) AND (“assumed benefits” OR “confirmed benefits” OR “benefit realisation management” OR “benefit realization management” OR “benefit realisation” OR “benefit realization” OR “benefit management”) AND (“human-centered design” OR “human centered design” OR “human-centred design” OR “human centred design” OR “user-centered design” OR “user centered design” OR “user-centred design” OR “user centred design”) AND (e-health OR ehealth OR “health information system” OR “healthcare information system” OR “hospital information system” OR “clinical information system*” OR “electronic health record” OR “digital health” OR “mobile health application” OR mhealth) AND (“assumed benefits” OR “confirmed benefits” OR “benefit realisation management” OR “benefit realization management” OR “benefit realisation” OR “benefit realization” OR “benefit management”) AND (e-health OR ehealth OR “health information system” OR “healthcare information system” OR “hospital information system” OR “clinical information system” OR “electronic health record” OR “digital health” OR “mobile health application” OR mhealth)

### Eligibility Criteria

Eligibility will be described by defining the criteria for including articles in the review and the criteria for excluding found publications from further processing. Studies will be selected according to the criteria described in Textbox 1.

**Eligibility criteria.**

**Inclusion criteria**

- Only peer-reviewed articles, conference proceedings, and book chapters.
- Papers that describe only quantitative, qualitative, or mixed methods.
- Papers that describe the use of human-centered design methodologies when designing digital health care solutions, such as co-design, cocreation, design sprint, or usability testing.
- Papers that describe the use of benefit realization management when developing digital health care solutions.
- Papers that describe if and how benefits have been realized with the help of human-centered design or benefit realization management when developing digital health care solutions.

**Exclusion criteria**

- Papers that are purely study protocols, systematic literature reviews, abstracts, posters, short papers, or scoping reviews.
- Papers that are not in English.
- Papers that are not research articles or publication or are otherwise off topic.
- Papers not related to digital health care solutions.
- If human-centered design or benefit realization management is explained but not applied in the digital health care solution.
- Papers that discuss fitness applications, well-being applications, and sport applications (we will only focus on applications and solutions related to therapeutic areas, such as cardiology, neurology, or oncology, that directly affect patient care, either through use by clinicians or together with patients’ care givers; therefore, in this review, we will exclude more consumer-targeted applications).

### Article Selection and Screening

After implementing the search strategy on the named databases, the results will be imported into Covidence (Veritas Health Innovation) to initiate the selection and screening process. Covidence automatically identifies duplicates and supports reviewers to execute their screening simultaneously and independently.

In the initial search and screening, the focus will be on titles and abstracts only. Three people will be involved in the screening. Each paper will be reviewed by at least 2 independent reviewers. All reviewers have backgrounds in digital health and information processing science. After completing the initial search and screening phase of the study, reviewers will move on to screening the full texts of the selected articles.

If the reviewers face disagreement in either the initial screening or during the full-text screening, reasons for disagreement will be discussed. If no resolution is found during the discussions, a fourth reviewer may act as an arbitrator to review the disagreed article and decide whether the article will be included or excluded. The disagreement will be documented together with the eventual outcome and its reason.

### Data Extraction and Analysis

The key information of the included articles will be extracted using the Data Extraction Form function available on Covidence. The extraction task will be completed by the 2 initial reviewers. The data extraction form within Covidence will assist in the extraction of relevant information from the selected studies.

### Assessment of Study Quality and Bias

Quality assessment will be produced in parallel to the data extraction process using a quality assessment form that will be included in Covidence. The quality assessment form will follow the checklist proposed by Dybå and Dingsøyr [17,18]. The checklist highlights the following main focus areas:

- The paper is based on research and research aims are clearly stated.
- Context, where research was carried out, is adequately described.
- The research design and recruitment strategy were appropriate for the aims of the research.
- The data were collected to address the research issues, and the data analysis was rigorous enough.
- The study is of value for research or practice.

### Data Synthesis

The included studies may be a collection of qualitative and quantitative data that will need to be transformed into a qualitative format. For data synthesis, narrative synthesis will be used, and therefore, a narrative form with an appropriate table format will be used. The table will consist of categories, such as the use of HCD, the use of BRM, evidence of assumed benefits (quantitative or qualitative), therapeutic areas (eg, cardiology, neurology, or oncology), modality of digital user interfaces (eg, desktop, mobile, or smartwatch), targeted user groups (eg, clinicians, patients, or care givers).

With the help of thematic coding, we will organize the extracted data into categories based on similarities and patterns. The categories will be further developed into a framework that will be used to construct a narrative addressing the proposed research questions and objectives of the review. Overall, the entire search strategy from the search and article selection to data synthesis will be piloted with 3-5 studies to address any need to change the approach.

## Results

As of January 2024, we have identified 2086 papers that have met our initial screening criteria. These papers are now being further analyzed to exclude those that do not exactly match our inclusion criteria described in Textbox 1.

## Discussion

The objective of this study specifically is to explore whether there is evidence that the use of HCD in the development of digital health care solutions helps the BRM process and enables better benefit realization.

Based on the initial search results and incomplete full paper screening, we can already see that there is evidence of using HCD in the creation of digital health care solutions, and by doing so, the benefits of the chosen design principle have been reported. However, the actual use of BRM in the context of digital health care solutions’ development does not seem so evident.

The large number of found papers in the initial search may be considered as a limitation of the study, potentially resulting in a broad sequence of alternative perspectives. We plan to critically review the inclusion and exclusion criteria while screening the remaining abstracts and titles and when reviewing selected full papers. This approach will increase the study’s quality level.

This review will enable the critical appraisal and synthesis of evidence on the successful use of HCD and BRM when developing digital health care solutions, leading to a better realization of the benefits set at the beginning of those projects. Controversially, if there is no evidence of both activities and causation, there is an opportunity for new knowledge creation going forward.

## Abbreviations

BM: benefits management
BRM: benefit realization management
HCD: human-centered design
PRISMA-P: Preferred Reporting Items for Systematic Review and Meta-Analysis Protocols
UCD: user-centered design
